# Population growth rate of dry bulb mite, *Aceria tulipae* (Acariformes: Eriophyidae), on agriculturally important plants and implications for its taxonomic status

**DOI:** 10.1007/s10493-017-0173-3

**Published:** 2017-08-30

**Authors:** Agnieszka Kiedrowicz, Brian G. Rector, Suzanne Lommen, Lechosław Kuczyński, Wiktoria Szydło, Anna Skoracka

**Affiliations:** 1 Population Ecology Lab, Faculty of Biology, Adam Mickiewicz University, Poznań, Poland; 2 Great Basin Rangelands Research Unit, USDA-ARS, Reno, NV USA; 3 Applied Plant Research, Wageningen UR, Wageningen, The Netherlands; 4 Department of Biology, University of Fribourg, Fribourg, Switzerland; 5 Department of Entomology, Institute of Agriculture and Natural Resources, University of Nebraska-Lincoln, Lincoln, NE USA

**Keywords:** Cryptic species, Host specificity, Host adaptation, Host range, Laboratory rearing, Garlic pest

## Abstract

Dry bulb mite (DBM), *Aceria tulipae*, is an economically important mite with a worldwide distribution and a broad host range. As a generalist, it is the most important eriophyoid mite attacking bulbous plants such as garlic, onion and tulip. To date, DBM has been recorded on host plants belonging to the families Liliaceae, Amaryllidaceae, Melanthiaceae and Asparagaceae. However, a precise understanding of DBM host range is lacking as it is largely based on casual records of mites on plants, some of which may include accidental hosts. Moreover, the possible existence of cryptic species has not been considered. In this study the hypothesis that DBM may be a complex of distinct genetic lineages or cryptic species was tested by comparing the common barcode sequence marker mtDNA COI of specimens from several populations originating from the Netherlands and Poland. The population growth rate of DBM on seven agriculturally important plant species and on various parts of the garlic plant was also experimentally assessed in the laboratory. The results did not support the first hypothesis, and indicated that DBM populations originating from Poland and the Netherlands shared essentially the same genome. In addition, they indicated that DBM reached the highest population growth rate on leek and also displayed high growth rates on garlic, chive and red onion, whereas white onion and wheat were not colonized by the mites. Answering the question of whether DBM is a single polyphagous species rather than a complex of cryptic lineages is of particular importance since the misidentification of pests may lead to ineffective control strategies. Moreover, improved knowledge of DBM host range is essential for assessing risk to crops.

## Introduction

Among eriophyoid mites, one of the most economically important is *Aceria tulipae* (Keifer), commonly known as the dry bulb mite (DBM), garlic mite, tulip mite or onion leaf mite (Navia et al. 2010). The DBM was originally described from specimens collected from tulip bulbs (*Tulipa gesneriana* L.) originating from the Netherlands (Keifer 1938). Since then, DBM has been recorded from many other plants in the families Liliaceae, Amaryllidaceae, Melanthiaceae and Asparagaceae, including both vegetables and ornamentals (Batchelor 1952; Conijn 1991; Conijn et al. 1996; MacLeod 2007). Early records of DBM infesting Poaceae (e.g. Slykhuis 1953; del Rosario and Sill 1965), however, were probably confounded with *Aceria tosichella* (Keifer), the wheat curl mite (WCM), infesting grasses such as wheat (*Triticum aestivum* L.) and quackgrass [*Elymus repens* (L.) Gould] (Skoracka et al. 2014).

The DBM is widespread around the world, with infestations recorded from 33 countries to date, representing all continents except Antarctica (Perring 1996; CABI 2006; Ostoja-Starzewski and Matthews 2006; Navia et al. 2010). As a host-generalist, it is the most important eriophyoid attacking bulbous crops (Conijn et al. 1996) including garlic (*Allium sativum* L.), onion (*Allium cepa* L.), and tulip (Leśna et al. 2014). It can cause severe losses to bulb crops, reducing yields up to 23% (Larrain 1986), and is an important problem for the Dutch tulip bulb industry. Most damage caused by DBM occurs while bulbs are in storage although the mite may also feed on growing leaves and flowers, and symptoms include bulb drying, as well as leaf twisting, curling and discoloration (Lange 1955; ChannaBasavanna 1966). The dry bulb mite is also a known vector of plant viruses such as *Tulip virus X* in tulips (Lommen et al. 2012), *Garlic mite*-*borne mosaic virus* (Garlic virus C) in garlic (Yamashita et al. 1996; Koo et al. 1998), *Onion mite*-*borne latent virus* in onion and *Shallot mite*-*borne latent virus* in shallot (van Dijk et al. 1991; Granda et al. 2017). It is considered as an invasive alien species in some countries, such as Japan (Mito and Uesugi 2004), Australia (Halliday and Knihinicki 2004), China (Hong et al. 2006), United Kingdom (Ostoja-Starzewski and Matthews 2006) and Iran (Khanjani and Haddad 2006). In addition to its great economic importance, there is increasing interest in *A. tulipae* as a study subject for basic and applied research. Experimental studies have focused on developmental time of DBM on garlic (Courtin et al. 2000), DBM infestation ability of various garlic varieties (Sapáková et al. 2012), methods of genetic identification of mites (Hein et al. 2012), impact of DBM on garlic production (Debnath and Karmakar 2013) and methods of biological control of DBM (Leśna et al. 2014). However, current knowledge of DBM host range is largely anecdotal.

An accurate understanding of DBM’s host range is essential for assessing risk to crops grown or stored in the vicinity of infestations. To date, such information has originated predominantly from records of its collection from various, mostly domesticated host plants (reviewed in Perring 1996). Such data, however, may include accidental hosts that do not support population growth and may overlook the existence of cryptic species as well as the possibility that a generalist herbivore may perform better on some host plant species than others (e.g. Skoracka and Kuczyński 2012).

The question of whether DBM may represent more than just a single polyphagous species is of particular importance since the misidentification of pests may lead to ineffective control strategies (Bickford et al. 2007). Although tulips are known as a primary host plant species for DBM, mite populations originating from onion and garlic in Poland did not successfully colonize tulip, whether in host-transfer or passive-infestation experiments (Skoracka et al. 2014). This suggests that DBM may be a complex of distinct genetic lineages or cryptic species differing in their host specificity. In this study we test this hypothesis by comparing DNA fragments of specimens from several populations originating from the Netherlands and Poland using the common barcode sequence marker mtDNA COI (Hebert et al. 2003). We also set out to experimentally assess the performance of DBM on several agriculturally important plant species in the laboratory, and on different parts of the garlic plant.

## Materials and methods

### Mite sampling

Tulip bulbs with DBM populations were obtained in October 2012 from post-harvest storage conditions from six locations in the Netherlands: Sint Maartensvlotbrug (52°46′45″N, 4°41′43″E), Enkhuizen (52°43′19″N, 5°16′07″E), Callantsoog (52°50′29″N, 4°42′30″E), Anna Paulowna (52°51′30″N, 4°48′17″E), Julianadorp (52°54′18″N, 4°44′21″E) and Lisse (52°15′19″N, 4°32′04.3″E). Tulip bulbs from each location were put into separate paper bags and sent to Poland, where they were hung separately in nylon bags on frames to provide air circulation and to avoid cross-contamination of mites between bags. These were maintained under ambient laboratory conditions (20–22 °C and 50–60% relative humidity) for 2 months to allow natural infestations of mites to proliferate. Some 5–20 mites were then collected from tulip bulbs from each locality and stored in 1.5-mL centrifuge tubes containing 180 μL of ATL buffer (Qiagen) at −20 °C until molecular identification was performed.

### DNA barcoding and analyses

Sequences from mite samples were obtained by isolating DNA using a nondestructive protocol, as described by Dabert et al. (2008). A fragment of the mitochondrial CO1 gene (ca. 630 bp of subunit I of the mitochondrial cytochrome c oxidase gene) was amplified by polymerase chain reaction (PCR) with the degenerate primers bcdF01 and bcdR04 (Dabert et al. 2008, 2010). PCR was carried out in 10-μl reaction volumes containing 5 μl of Type-it Multiplex PCR Master Mix (2xMM) (Qiagen), 0.5 μM of each primer, and 4 μl of DNA template. The thermal cycling profile consisted of: an initial step of 5 min at 95 °C; 35 cycles of 30 s at 95 °C, 1 min at 50 °C, and 1 min at 72 °C; and a final step of 5 min at 72 °C. The PCR products were diluted by half before agarose-gel (1%) electrophoresis. Products that showed clear bands of appropriate size were sequenced (both strands) with the same primers as were used for amplification. Sequencing was performed with BigDye Terminator v.3.1, in accordance with the manufacturer’s protocol and products from the sequencing reaction were analyzed on an ABI Prism 3130XL (Applied Biosystems). Trace files were checked and edited using MEGA v.6 (Tamura et al. 2013). Sequences have been deposited in GenBank under accession numbers: KY610210-KY610215.

Sequences of Polish DBM populations from garlic and onion that were obtained from the GenBank database under accession numbers FJ387563 and JF920096 were also included in the analysis. CO1 sequences were aligned by CLUSTAL W using MEGA v.6 (Tamura et al. 2013) with default gap weighting parameters, followed by manual adjustment. Alignment of COI sequences was trimmed to 604 bp and checked by translating the aligned DNA into amino acid sequences. Overall and pairwise distances between nucleotide sequences were calculated using Kimura’s two-parameter model (Kimura 1980) (all codon positions and pairwise gap deletion) and standard errors were calculated with 1000 bootstrap replicates.

### Mite cultures

Since tulip bulbs desiccated during storage, we decided to transfer the mite cultures onto garlic in December 2012. Because mtDNA COI sequences of all tulip populations were almost identical genetically (see “Results” section) we established a mixed culture of mites from tulips from all populations. Before transferring to garlic, we determined an optimal method of rearing DBM on garlic by transferring 10 females from tulip populations onto different parts of mite-free garlic (‘treatments’ hereafter): (1) cloves with husks, (2) cloves without husks (peeled garlic) and (3) green leaves of growing garlic, establishing at least six replicates for each treatment. Shortly after mite transfer, the number of females that had successfully settled on the test material was recorded and those that had been injured or had died were removed. Each replicate was put in a separate rearing cage on a petri dish covered by a nylon bag (for cloves) or in metal frames covered by nylon cloth (for growing garlic plants) and maintained under laboratory conditions (20–22 °C and 50–60% r.h.) for 14 days. All replicates were then visually inspected under a stereomicroscope to count all mites, which involved the destruction of plant material to ensure that all mites inside the plant structures were found. As this preliminary experiment indicated that population growth rates would be highest on garlic cloves without husks (see “Results” section), we established stock populations on such cloves. Garlic cloves were inspected every 2 weeks and old, rotten or dried garlic cloves were replaced with fresh ones.

### Host specificity

Survival and reproduction of DBM was tested and quantified on the foliage of six crop plant species: garlic; bulb onion, both white and red; shallot; leek; chive (all Alliaceae); and wheat (Poaceae). Green leaves of growing plants were tested; these were grown from either mite-free bulbs (garlic, onion, shallot) or seeds (leek, chives, wheat) in commercial potting soil. Plants were grown in rearing cages for 2 weeks (wheat), 4 weeks (garlic, onion, shallot and chives) or 24 weeks (leek) until the appropriate stage for infestation by mites was obtained. Some 10–15 DBM females were transferred under a stereomicroscope from the stock population onto clean plants in 6–15 replicates, using an eyelash glued to a dissection needle. Shortly after transfer, the number of females that had successfully settled on the test plants was counted, and those females that had been injured or died during transfer were removed. Test plants were put into rearing cages (metal frames wrapped with nylon cloth) and maintained under ambient laboratory conditions for 14 days. Afterwards, test plants were inspected under a stereomicroscope and mites were counted on entire plants (green leaves and bulbs, if present). The test plants were destructively dissected to ensure that all mites were found.

### Statistical analysis

A population growth rate statistic (*r*) was used as a measure of the colonization ability of DBM in the experiments. It was defined according to the formula: *r* = log_2_[(*n*/*n*_0_) + 1], where *n* represents the number of specimens found after 14 days after the start of each experiment and *n*_0_ represents the number of settled females shortly after transfer. The interpretation of this index is: if *r* = 1, the population size did not change (i.e. none of the females reproduced but all survived, or the same number of mites died as were born); if *r* > 1, the population increased, indicating successful reproduction; if *r* < 1, the population decreased (i.e. females did not reproduce or the reproduction rate was lower than mortality rate). When *r* = 0, the whole population was extinct (i.e. no specimens alive after 14 days) (Skoracka et al. 2014). Mean population growth rates were calculated for each treatment separately. Ninety-five-percent confidence intervals (95% CI) around the means of *r* were calculated using bias-corrected and accelerated bootstrap (Efron and Tibshirani 1993). To test whether *r* differed significantly between treatments, a simple one-way ANOVA was performed. Post-hoc paired comparisons of mean population growth rates were conducted using least significant difference (LSD) values at the 0.05 level of significance. R v.3.0.2 was used for all analyses (R Development Core Team 2013).

## Results

### Barcoding

Sequences (604 bp) obtained for the studied populations were almost identical; the overall alignment of six sequences from the Netherlands and two sequences from Poland had only three variable sites. Mean overall K2-P distance was 0.2% (SE = 0.1%), whereas pairwise distances between nucleotide sequences ranged from 0.0 to 0.5% (SE = 0–0.3%).

### Culturing on different garlic parts

**Fig. 1:**
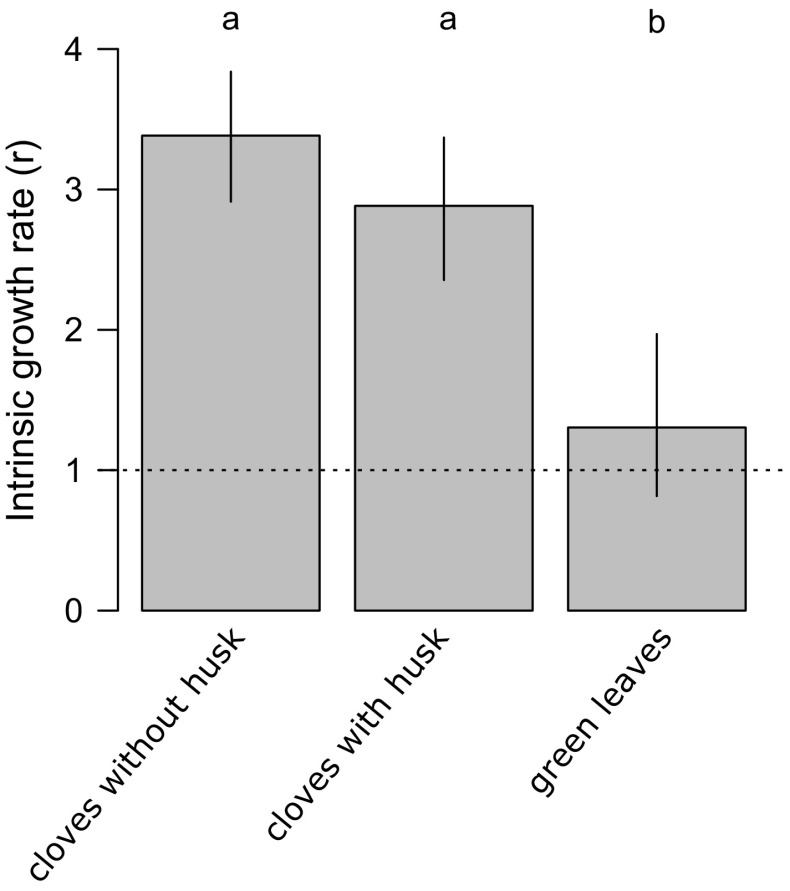
Population growth rates (*r*) of the dry bulb mite transferred from tulip bulbs to garlic (garlic cloves without husks; garlic cloves with husks; green leaves of sprouted garlic). Bootstrapped 95% confidence intervals around means of *r* are shown. The *broken line* (*r* = 1) indicates no change in population size. Treatments with the *same letter* were not significantly different (least significant difference test)

The colonization of all three garlic plant parts was successful, but the population growth rates differed significantly (F_3,38_ = 93.4, *p* < 0.0001). Population growth was significantly lower on green leaves (*r* = 1.3, 95% CI 0.8–2.0) compared to garlic cloves with husks (*r* = 2.9, 95% CI 2.3–3.7) or without husks (*r* = 3.4, 95% CI 2.9–3.8). The highest DBM population growth rate was observed on garlic cloves without husks although the difference was not significant when compared to garlic cloves with husks (Fig. 1).

### Host specificity

**Fig. 2:**
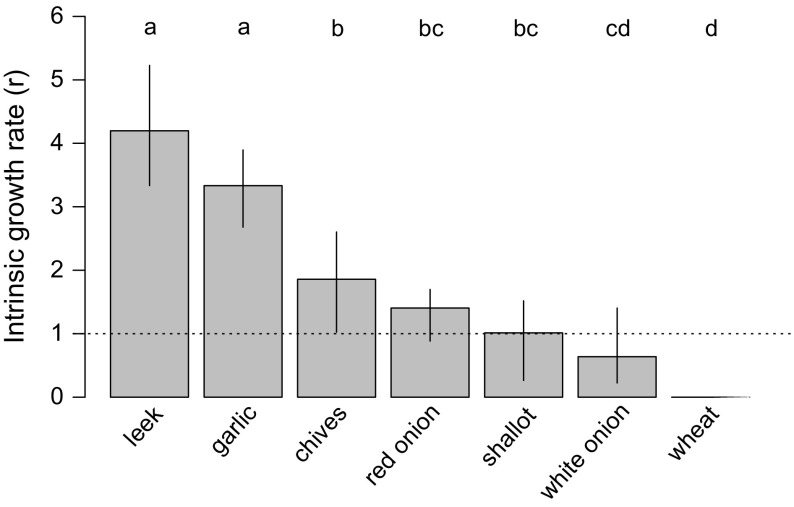
Population growth rates (*r*) of the dry bulb mite engaged in host colonization tests on seven crop plant species (leek, garlic, chives, red onion, shallot, white onion and wheat) when transferred from garlic cloves without husks. Bootstrapped 95% confidence intervals around means of *r* are shown. The *broken line* (*r* = 1) indicates no change in population size. Treatments with the *same letter* were not significantly different (least significant difference test)

Population growth differed significantly among the crop plants tested (F_7,35_ = 42.4, *p* < 0.0001). The DBM successfully reproduced on leek (*r* = 4.2, 95% CI 3.3–5.2), garlic (*r* = 3.3, 95% CI 2.7–3.9), chives (*r* = 1.9, 95% CI 1.0–2.6), and red onion (*r* = 1.4, 95% CI 0.9–1.7). On shallot, population size did not change (*r* = 1.0, 95% CI 0.3–1.5). By contrast, the population growth rate on white onion was below the threshold (*r* = 0.6, 95% CI 0.2–1.4), and no survival was obtained on wheat (*r* = 0). Population growth rate of DBM was highest on leek and garlic (Fig. 2).

## Discussion

Taking into account the differences in host acceptance by dry bulb mite (DBM) populations, genetic variation within this species was expected (Skoracka et al. 2014). However, our results did not support the hypothesis that DBM consists of distinct genetic lineages or cryptic species. The mtDNA COI sequences originating from Dutch and Polish DBM indicated a lack of genetic differentiation between populations from these countries. Differences between sequences were less than 1%, a value indicating intra-specific variation (Bickford et al. 2007; Skoracka et al. 2015). A mean interspecific COI sequence divergence of 11.2% (using the Kimura-two-parameter model) was calculated for more than 13,000 congeneric pairs of various animals (Hebert et al. 2003), whereas values of interspecific COI divergence have ranged from 4.9 to ca. 20% in several species of mites (Anderson and Morgan 2007; Dabert et al. 2008; Tixier et al. 2008; Lewandowski et al. 2014; Cvrković et al. 2016). Thus, the results presented here (viz. almost identical COI sequences among the tested DBM populations) suggest the lack of genetic differentiation, at least in the COI barcode between the tested populations. However, to validate this conclusion more extensive testing, including a wider spectrum of molecular tools, would be necessary.

The results of our study demonstrate the ability of DBM to infest several agriculturally important plants, such as leek, garlic, chives, red onion and shallot. Since DBM is frequently recorded as a serious pest of onion and garlic (Ostoja-Starzewski and Matthews 2006; Navia et al. 2010; Debnath and Karmakar 2013), we predicted that in our assays mites will reach their highest population growth rate on these two plant species. Contrary to our expectations, DBM reached its highest population growth rate on leek (Fig. 2). The DBM may therefore represent a threat to leek cultivation, especially for young seedlings produced in greenhouses. In the literature, DBM is not commonly reported as a pest of leek, perhaps because this plant is grown more often in the field than in greenhouses, and environmental conditions in leek fields may be unfavorable for DBM.

White onion was not successfully colonized by Dutch populations of DBM (Fig. 2), and this poor performance is striking because bulb onion (and white onion in particular) is often mentioned as a typical host for DBM (Liro 1942; Keifer 1946, 1952; Perring 1996; Hein et al. 2012). Moreover, a Polish DBM population colonized a different onion variety (including white) with great success (Skoracka et al. 2014). This may suggest the existence of genetic diversity within *A. cepa* for susceptibility to DBM, as was shown for various garlic varieties in Czechia (Sapáková et al. 2012); this possibility merits further investigation. Moreover, the population growth rate on red onion was lower in our studies when compared to results reported by Skoracka et al. (2014). Although the population growth rates on white and red onion were not significantly different in the study presented here, these were tested on green leaves, whereas Skoracka et al. (2014) tested population growth rate on bulbs. The results of the preliminary experiments indicated that DBM achieved higher population growth rate on garlic cloves than on green leaves, so it may be similar in the case of onion. Our results indicated that the Dutch populations of DBM did not survive on wheat (Fig. 2), which is consistent with results obtained by Skoracka et al. (2014).

DBM achieved higher population growth on garlic leaves when transferred from stock colonies maintained on garlic for several generations (Fig. 2; *r* = 3.3, 95% CI 2.7–3.9) compared to transfer from the original populations on tulip (Fig. 1; *r* = 1.3, 95% CI 0.8–2.0), which may suggest that garlic is a more suitable host when compared to tulip. It has been shown that some host plants may create various chemical, physical, and phenological conditions, which can have a great influence on the host acceptance decisions and the life history traits of generalist herbivore species, which further influence species abundance on the plant (Jaenike 1990; Agrawal et al. 2002; Chapman 2003). An alternative explanation is that we observed the adaptation of the stock colony population to the host plant species over the course of several generations. One consequence of such host adaptation may be the reduction of gene flow between populations associated with different hosts or microhabitats (Futuyma and Moreno 1988; Brooks and McLennan 1993; Hebert et al. 2004; Steinauer et al. 2007). This may also explain the results obtained by Skoracka et al. (2014): viz. adaptation of Polish DBM populations to garlic and onion may have resulted in selection against acceptance of tulip bulbs. Thus, we may also expect strong host adaptation to plants belonging to different families (e.g. Liliaceae vs. Amarylidaceae).

It is possible that early records of DBM from different hosts belonging to the families Liliaceae, Amaryllidaceae, Melanthiaceae and Asparagaceae (Batchelor 1952; Conijn 1991; Conijn et al. 1996; MacLeod 2007) were confounded with the wheat curl mite (WCM) complex, since these two eriophyid mites are very similar morphologically (despite differences in body size) and some WCM lineages (e.g. WCM MT-1) can survive on garlic and onion when transferred from wheat (Skoracka et al. 2014). Verification of these host records and additional assessments of DBM colonization of wild members of these families is warranted, as such wild species could act as reservoirs for this pest and increase its potential for spread. It would be necessary to perform detailed studies, including field experiments (e.g. surveys for DBM on plants from the aforementioned families), host-acceptance experiments in the laboratory and molecular identification of specimens in order to establish a full and accurate host range of *Aceria tulipae* sensu lato.

## Conclusions

The results of this study demonstrate differences in the ability of DBM to infest different cultivated plants belonging to the families Alliaceae and Poaceae. Data on the host specificity of various DBM populations are essential to estimate their respective threats to crop production and their invasive potential, allowing development of control strategies for this pest mite species. Moreover, this study has advanced basic research on this species, providing new data on methods of efficiently rearing DBM. In addition, it was found that DBM populations from Poland and the Netherlands have very similar mtDNA COI sequences suggesting that, in spite of the observed differences in their host performance (results in this study and Skoracka et al. 2014), they are closely related members of a single species. Future studies of DBM host specificity (e.g. testing a wider range of host plant species) and DBM population genetic structure are warranted.
